# Pharmacodynamic modelling of in vitro activity of tetracycline against a representative, naturally occurring population of porcine *Escherichia coli*

**DOI:** 10.1186/s13028-015-0169-0

**Published:** 2015-11-24

**Authors:** Amais Ahmad, Camilla Zachariasen, Lasse Engbo Christiansen, Kaare Græsbøll, Nils Toft, Louise Matthews, Peter Damborg, Yvonne Agersø, John Elmerdahl Olsen, Søren Saxmose Nielsen

**Affiliations:** Department of Large Animal Sciences, Faculty of Health and Medical Sciences, University of Copenhagen, Frederiksberg C, Denmark; Department of Veterinary Disease Biology, Faculty of Health and Medical Sciences, University of Copenhagen, Frederiksberg C, Denmark; Department of Applied Mathematics and Computer Science, Technical University of Denmark, Lyngby, Denmark; National Veterinary Institute, Section of Epidemiology, Technical University of Denmark, Frederiksberg C, Denmark; Boyd Orr Centre for Population and Ecosystem Health, College of Medical, Veterinary and Life Sciences, University of Glasgow, Glasgow, UK; Department of Epidemiology and Microbial Genomics, Technical University of Denmark, Lyngby, Denmark

**Keywords:** Antimicrobial resistance, Tetracycline, Pharmacodynamic, Pig

## Abstract

**Background:**

The complex relationship between drug concentrations and bacterial growth rates require not only the minimum inhibitory concentration but also other parameters to capture the dynamic nature of the relationship. To analyse this relationship between tetracycline concentration and growth of *Escherichia coli* representative of those found in the Danish pig population, we compared the growth of 50 randomly selected strains. The observed net growth rates were used to describe the in vitro pharmacodynamic relationship between drug concentration and net growth rate based on *E*_*max*_ model with three parameters: maximum net growth rate (*α*_*max*_); concentration for a half-maximal response (*E*_*max*_); and the Hill coefficient (γ).

**Results:**

The net growth rate in the absence of antibiotic did not differ between susceptible and resistant isolates (P = 0.97). The net growth rate decreased with increasing tetracycline concentrations, and this decline was greater in susceptible strains than resistant strains. The lag phase, defined as the time needed for the strain to reach an OD_600_ value of 0.01, increased exponentially with increasing tetracycline concentration. The pharmacodynamic parameters confirmed that the $\alpha_{max}$ between susceptible and resistant strains in the absence of a drug was not different. *EC*_*50*_ increased linearly with MIC on a log–log scale, and γ was different between susceptible and resistant strains.

**Conclusions:**

The in vitro model parameters described the inhibition effect of tetracycline on *E. coli* when strains were exposed to a wide range of tetracycline concentrations. These parameters, along with in vivo pharmacokinetic data, may be useful in mathematical models to predict in vivo competitive growth of many different strains and for development of optimal dosing regimens for preventing selection of resistance.

## Background

Resistance to antimicrobials is a continuous challenge to the public health care system [1]. In vitro growth response of bacterial strains to antimicrobial is required to evaluate carefully before designing treatment protocols for antimicrobial use. Tetracycline is a widely used broad-spectrum antimicrobial that inhibits the growth of many bacteria. It is the most commonly used drug against gastrointestinal infectious diseases in pig production in Denmark, a production system that accounted for 76 % of the total veterinary consumption in kg active compound in 2012 [2]. It is also used in humans, with 11 % of the total consumption of antimicrobials being used in primary health care in Denmark in 2012 [2]. Tetracyclines exert concentration and time-dependent antimicrobial effects [3]. After binding to the ribosome, tetracyclines inhibit the binding of aminoacyl-tRNA to the messenger RNA molecule/ribosome complex, thereby interfering with bacterial protein synthesis in growing or multiplying organisms [4]. Tetracycline resistance is generally due to the acquisition of genes [5, 6], encoding either energy dependent efflux proteins, which transfer tetracyclines out of the bacterial cell, ribosomal protection proteins, which make the ribosome insensitive to tetracycline inhibition by interacting with the tetracycline binding site(s), or enzymes, which deactivate tetracyclines in the presence of oxygen [7].

*Escherichia coli* is commonly used as an indicator bacterium for antimicrobial resistance in animals, humans, and food products [2]. In Denmark, 36 % of commensal *E. coli* obtained from pigs were resistant to tetracycline, and efflux pumps TetA and TetB were found to encode for resistance in a subset of *E. coli* isolates from 2012 [2, 8]. Such a high level of resistance is worrisome considering the widespread use of tetracyclines against diseases (e.g. post-weaning diarrhoea) where *E. coli* is often involved. Furthermore, resistance may spread horizontally to other bacterial species in the gut microbiota. Understanding the relation between tetracycline exposure and the growth response of porcine *E. coli* strains is important to aid in improving dosing strategies and possibly reduce the resistance problem.

Growth response of bacterial strains exposed to a specific antimicrobial agent is usually evaluated based on pharmacodynamics (PD) of antimicrobials. In such studies, strains are commonly characterized by the PD parameter minimum inhibitory concentration (MIC), defined as the lowest antibiotic concentration that prevents visible growth of the bacterial population in vitro [9–11]. This parameter is measured only at one time point after exposure of the bacterial population to a constant antimicrobial concentration, and it does not reflect the time-inhibition process. A more useful approach is to base in vitro models on time kill curves [12, 13] using a modelling approach where the relationship between estimated growth rates and antimicrobial concentrations is analysed by maximum-effect (*E*_*max*_) models. Such *E*_*max*_-models are based on three key PD parameters: maximum growth rate, i.e., growth without antimicrobial pressure, drug concentration leading to a half-maximum effect (*EC*_50_); and the Hill’s coefficient, describing the steepness of a sigmoid Hill equation [9, 10]. These parameters along with in vivo pharmacokinetics (PK) have been used as an input for the mathematical models of in vivo bacterial growth [14–18]. These in vivo models have been used to propose new treatment strategies [19, 20]. Inference based on the in vivo model predictions can be used to design treatment strategies to, helping reduce costly clinical studies and the use of experimental animals [19, 21].

The aim of the current study was first to investigate the relation between tetracycline concentration and growth performance of a representative collection of commensal intestinal strains of *E. coli* and second to estimate PD parameters and their association with MIC values in the presence of increasing concentrations of antibiotics using an in vitro PD model.

## Methods

### Strain selection

In 2010, 160 porcine commensal *E. coli* isolates were obtained from the same number of healthy pigs from different herds at slaughter as part of the Danish Integrated Antimicrobial Resistance Monitoring and Research Program (DANMAP) [22]. Among these isolates, 50 were selected randomly for this study. The selection was performed using a computerized random sample generator, and it was considered to be representative of the Danish pig population. Isolates were confirmed as *E. coli* by matrix-assisted laser desorption ionization-time of flight (MALDI-TOF) mass spectrometry [23] using E. coli ATCC 8739 as reference strain and SaramisTM 3.5 (bioMérieux) for spectra interpretation.

### Susceptibility to tetracycline

The susceptibility to tetracycline was tested in two-fold dilutions between 0.125 and 512 µg/ml tetracycline hydrochloride (Sigma-Aldrich, Switzerland) for all 50 isolates using broth microdilution following the Clinical and Laboratory Standards Institute (CLSI) standards [24]. Briefly, two-fold dilutions of tetracycline were prepared in Cation-Adjusted Mueller-Hinton 2 broth (MH-2) (Sigma-Aldrich, Switzerland) and distributed on microtitre plates. Bacterial saline suspensions were prepared from overnight cultures on blood agar and adjusted to a 0.5 McFarland turbidity standard. The suspensions were diluted 1:100 in MH-2 and this suspension was used as inoculum of the wells, giving a final concentration of approximately 5 × 10^5^ CFU/ml. *E. coli* ATCC®25922 was used as a quality control. Isolates with MIC ≥16 µg/ml tetracycline were considered to be tetracycline resistant [25].

### Growth curves

Growth curves for the effect of tetracycline on the 50 *E. coli* strains were obtained using the automated microbiology growth curve analysis system BioScreen C^TM^ (Oy Growth Curves Ab Ltd, Finland). Two-fold dilutions of tetracycline hydrochloride were distributed into BioScreen plates. Bacterial saline suspensions were prepared from overnight cultures on blood agar and adjusted to a 0.5 McFarland turbidity standard. These suspensions were diluted 1:100 in MH-2, and inoculated to the plates to give a final concentration of approximately 5 × 10^4^ CFU/ml. Final volume in each well was 200 µl. Susceptible isolates were grown in two-fold dilutions of tetracycline ranging from 0.03 to 8 µg/ml, and resistant isolates were grown in two-fold dilutions ranging from 0.5 to 128 µg/ml of tetracycline. All isolates were additionally grown in MH-2 without antibiotics. The BioScreen was set to 18 h incubation at 37 °C with continuous shaking and optical density (OD) at 600 nm measured every 5 min. All experiments were performed in biological triplicates, meaning that bacterial culture, adjustment of cell density, preparation of tetracycline suspensions, and running on the BioScreen were done three times for each tested strain. Strains that do not grow because of MIC above the concentration tested were assigned a growth rate of zero.

### Analysis of growth curves

The BioScreen raw data were extracted in Microsoft Excel. OD values of blank samples were subtracted from sample OD values at the respective time points before analysing the data.

The effect of tetracycline on growth of *E. coli* was assessed from the net growth rate (*µ*) of the strains at various tetracycline concentrations. The linear relationship between CFU and OD is only valid for low cell concentrations, and this relation becomes unreliable above a certain critical value [26, 27]. An OD of 0.1 was taken as a maximum reliable value in this study. Exponential growth part of growth curves below this cut-off was used for the model fit. The following model equation was used:

1 $$ Y_{t} = \lambda e^{\mu t} + \beta + \varepsilon_{t} $$

where $Y_{t}$ is the OD value, *λ* is the initial OD value at time zero,* µ* is the growth rate, *β* is an offset variable for the adjustment of *λ*, and $\varepsilon_{t}$ normal error with mean zero and constant variance $\sigma^{2}$; i.e., $\varepsilon_{t}$ = N (0, $\sigma^{2}$). Growth rates for the 50 *E. coli* strains at each concentration level were estimated by fitting the model (Eq. 1) to growth curves using a nonlinear least square algorithm nls() function of the R statistical software [28].

The lag phase could not be identified from the OD results using the BioScreen. To get an indication of the effect of tetracycline on growth onset of *E. coli*, the time needed for the different strains to reach an OD value of 0.01 at the various tetracycline concentrations was analysed. Growth onset values were determined as the first time value at which OD was equal to or slightly greater than 0.01. Because the growth onset between triplicates can be influenced by external factors, growth onset relative to growth onset when grown without tetracycline was calculated for each triplicate and tetracycline concentration.

A Kruskal–Wallis one-way analysis of variance and Wilcoxon rank-sum test was used to compare the growth rates and relative growth onset between tetracycline susceptible and resistant strains at various tetracycline concentrations using R [28]. The level of significance was set at P < 0.05.

### Pharmacodynamic model

The relationship between the tetracycline concentration and the estimated net bacterial growth rates was analysed using the sigmoid $E_{max}$ model [17, 29–31]. The net *E. coli* growth rate as a function of drug concentration *c* was described previously [17]:

2 $$ \alpha {\mkern 1mu} (c) = \alpha _{{{\text{max}}}}  - \frac{{\alpha _{{{\text{max}}}} \left( {\frac{c}{{EC_{{50}} }}} \right)^{\gamma } }}{{1 + \left( {\frac{c}{{EC_{{50}} }}} \right)^{\gamma } }}  $$

where $\alpha_{max}$ is the bacterial growth rate in the absence of the drug (maximum effect), $EC_{50}$ is the concentration at which the drug effect is reduced to 50 %, and γ denotes the Hill coefficient, which is the measure of the steepness of the sigmoid relationship between concentration *c* and the growth rate at concentration *c*. Growth rates in triplicates derived from the exponential growth model (Eq. 1) were plotted against the concentration range and fitted to the model given by Eq. 2 for each of the 50 *E. coli* strains, using a nonlinear least square algorithm nls() function of R [28]. PD model parameters $\alpha_{max}$ and γ were compared for susceptible and resistant strains using the Wilcoxon rank-sum test in R, whereas the linear relation between MIC and $EC_{50}$ was analysed using the lm() function in R.

## Results

### Tetracycline MIC distributions

All isolates were verified as *E. coli* by MALDI-TOF MS analysis. Antibiotic resistance patterns of the 50 randomly selected isolates had previously been determined as part of the Danish Integrated Antimicrobial Resistance Monitoring and Research Programme 2010, however, only for tetracycline concentrations between 2 and 32 µg/ml [22], and therefore susceptibility towards this drug was examined in more detail in the current study. Among the 50 isolates, seventeen (34 %) were resistant to tetracycline with an MIC ≥16 µg/ml.

### Growth response to different concentrations of tetracycline

**Fig. 1:**
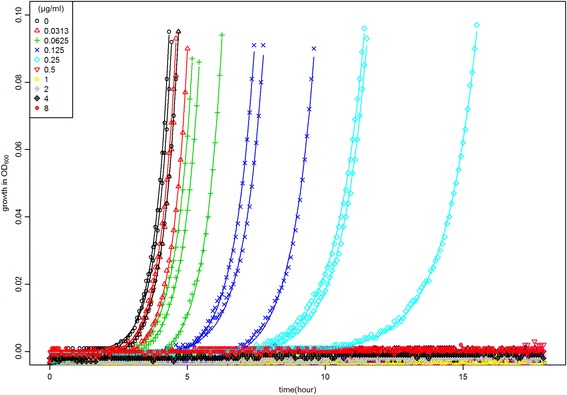
Growth curves of a strain with MIC = 0.5 µg/ml under range of tetracycline concentrations with maximum OD value of 0.1. Each colour represents growth in triplicates at a specific concentration

Growth curves of a single *E. coli* in triplicate at different concentrations of tetracycline are shown in Fig. 1, with an exponential model fit indicated by the solid lines. The drug showed a clear inhibition effect with increasing concentrations, whereas growth onset was shown to increase with increasing concentrations of the antibiotic.

**Fig. 2:**
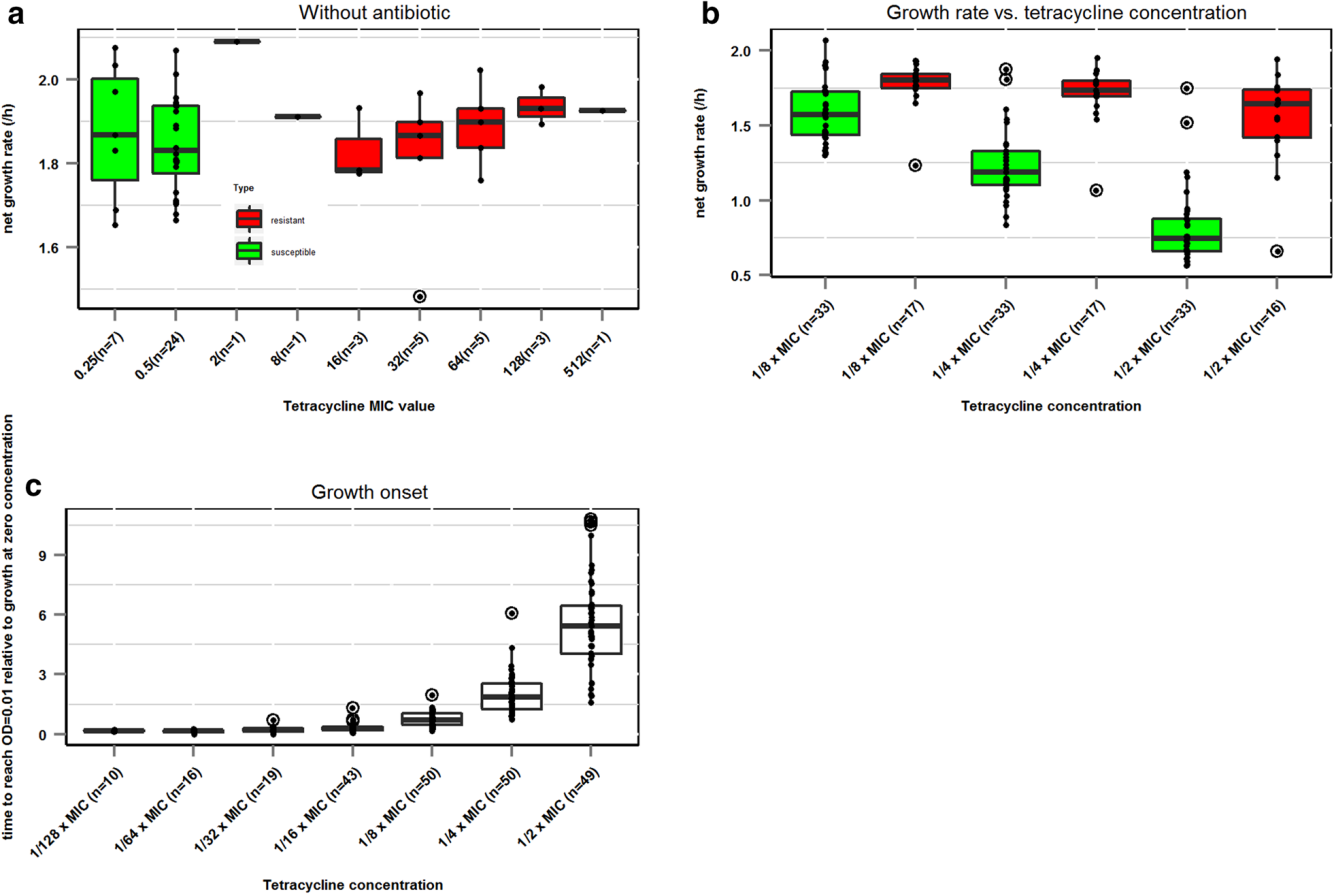
**a** Net growth rate (h^−1^) of the isolates in the absence of tetracycline, grouped according to tetracycline MIC. **b** Net growth rate (h^−1^) of tetracycline susceptible and resistant isolates grown in increasing concentrations of tetracycline. **c** Relative growth onset of the isolates when grown in increasing concentrations of tetracycline. **a, b** *Green box plots* refer to estimated net growth rates of tetracycline susceptible isolates, red boxplots to estimated net growth rates from tetracycline resistant isolates. *Black dots* indicate mean net growth rate (h^−1^) of the individual isolates. **a**–**c** Outliers are shown as *circled dots*. The number of isolates in each group is indicated in *brackets*

In the absence of tetracycline, only minor insignificant variations between the net growth rates of the individual *E. coli* strains were found, indicating a lack of fitness cost, and no significant correlation was found between the MIC-values and growth rates (Fig. 2a). When analysed as groups, the net growth rates of tetracycline susceptible and resistant isolates did not differ in the absence of antibiotics, however at tetracycline concentrations corresponding to ^1^/_2_, ^1^/_4,_^1^/_8_ of the MIC value of the strains, the growth rates were significantly less affected in the resistant isolates than the susceptible ones (Fig. 2b). Overall, the effect of sub-MIC concentrations of tetracycline on onset of growth (time to reach OD = 0.01) increased exponentially with increasing tetracycline concentration (Fig. 2c). When expressed within sub-MIC group, the effect of the concentration of drug on onset of growth was not statistically significant at any of the tetracycline concentrations between tetracycline susceptible and resistant strains (data not shown).

### Pharmacodynamic modelling

**Fig. 3:**
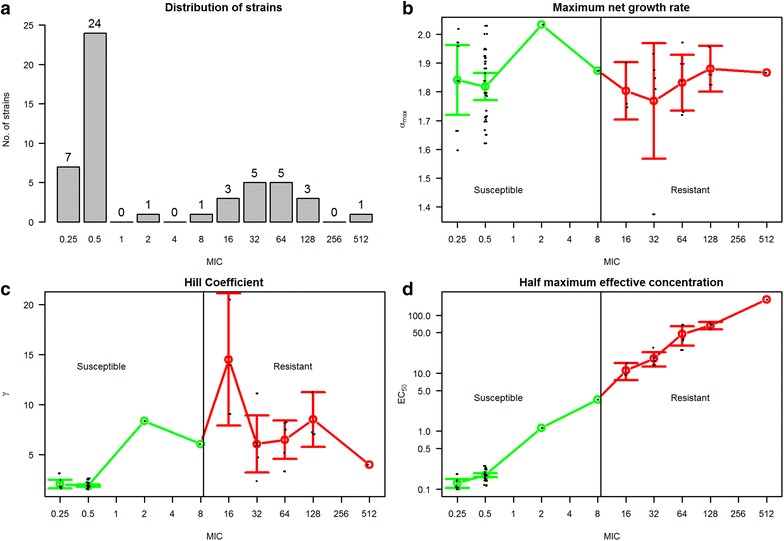
**a** Distribution of 50 *E. coli* strains according to MIC values. **b**–**d** Three PD parameters from *E*_*max*_ model of 50 *E. coli* strains according to their MIC values with mean and 95 % confidence interval. The *green line* represents susceptible isolates and the *red line* represents resistant isolates. A *vertical black line* is drawn at cut-off between susceptible and resistant isolates

The MIC distribution of strains is presented in Fig. 3a. Model parameter $\alpha_{max}$ did not differ significantly (P = 0.97) between resistant and susceptible strains (Fig. 3b). In contrast, the Hill coefficients (γ), capturing the steepness of response to tetracycline, were smaller for susceptible strains than resistant strains (P < 0.00001; Fig. 3c), showing that the susceptible strains were more affected by low concentrations of drugs than resistant ones. There was a significant linear relationship between MICs and *EC*_*50*_ values on the log–log scale (P < 0.00001; Fig. 3d) with an R^2^ of 0.99.

## Discussion

This is to our knowledge the first large-scale study investigating the effect of tetracycline on growth in *E. coli* obtained randomly from healthy pigs in different farms. The study was based on in vitro growth curves, which were used to fit an in vitro PD model for the inhibitory effect of tetracycline. This approach greatly increases the possibility of including many strains and many concentrations of antibiotics. In contrast, previous studies that have characterized the growth effect of antimicrobials on *E. coli* were based on manual growth curves for a limited number of strains, most often of clinical origin [32–35]. Clinical *E. coli* are more likely to be resistant than commensal strains [2], thus our decision to include a large number of commensal strains illustrates better the full diversity of MIC-distributions present in *E. coli* at herd level.

We focused on the differences in activity of tetracycline against tetracycline susceptible and resistant *E. coli* using real time growth curves. Growth curves were fitted, and in vitro PD parameters were derived using the *E*_*max*_ model by fitting the relation between concentration and net bacterial growth rates.

We did not find any association between growth rates and MIC values in the absence of tetracycline, i.e. the maximum net growth rate *α*_*max*_ (growth in the absence of drug) was found to have no significant difference between susceptible and resistant strains. The lack of fitness costs associated with tetracycline resistance might be explained by the mechanism by which tetracycline resistance genes are regulated.

Basing the study on growth performance in BioScreen had some limitations. A true relation between colony forming units and OD values was unknown, and a linear relation was assumed with a maximum OD value of 0.1. Growth onset was defined as the time needed for the cells to reach the exponential growth phase following the lag phase. We defined growth onset as the time for a strain to reach an OD value of 0.01, as preliminary experiments showed this OD value to be part of the early exponential phase (data not shown). Future experiments with manually obtained growth curves in flasks could overcome these limitations, but the advantage of being able to include many strains in the study was considered the critical goal.

*EC*_*50*_ was highly correlated with the MIC, showing a strong relationship between this point estimate parameter and the dynamic activity of tetracycline, i.e. the antimicrobial concentration required to produce 50 % of the maximum effect *EC*_*50*_ was found to increase linearly with MIC values on a log–log scale. A likely explanation is that MIC reflects a difference between strains in the ability to pump out tetracycline and that this inherent difference between strains is not dependent on concentration of tetracycline. In that situation, a strain that is superior at one concentration of drug will also be superior at another concentration. It should be noted that *EC*_*50*_ values occasionally differed among strains with similar MIC values, but this may be explained by methodological shortcomings. For example, the inoculum density in the BioScreen setup was slightly lower than the density used for MIC testing. Furthermore, MIC values are associated with uncertainty (two fold) and some degree of subjectivity, whereas the OD-based values here are recorded objectively using BioScreen C^TM^. Also, in the BioScreen experiments the strains were grown with constant shaking, as opposed to the MIC assay without shaking. Constant shaking makes *E. coli* grow faster due to the aeration of the culture supplying the bacteria with plenty of oxygen. There is also variation associated with the *EC*_*50*_ estimates, but to a lower degree, as it is (generally) well-defined within two tested concentrations.

The steepness of response to tetracycline concentrations was found to be greater in the case of resistant strains, but estimates were associated with large uncertainty. Thus, this result should be viewed with more caution than other, since relatively high values of γ in resistant strains could be due to the fewer data points around the *EC*_*50*_. Additional in vitro growth experiments were performed including tetracycline concentrations between some of the higher two-fold dilutions, in order to overcome this problem (data not shown). Still, it was not possible, within the limitations of the study, to obtain as many data points for the high MIC values as for the low ones.

## Conclusions

This study based on in vitro growth experiments of the complex relationship between *E. coli* growth and tetracycline concentration showed that along with MIC values, there are other parameters that govern this relationship. These parameters should also be taken into account in addition to the MIC when studying the bacterial response to antimicrobials and were described and estimated in this study using pharmacodynamic modelling. These parameters better capture the dynamic activity of a drug, for which point estimate MIC cannot account, and this may affect the in vivo outcomes. The estimated in vitro PD population based parameters may be used along with in vivo pharmacokinetics in mathematical models for predicting the in vivo growth of multiple strains. Predictions from these in vivo models may be useful in optimizing dosing regimens for suppressing selection of resistance. Although this study did not identify fitness costs, further work to characterize the potential impact of fitness costs in a population based setting is needed, since the presence of fitness costs is a commonly-made assumption in research on antimicrobial resistance, in particular in relation to the disappearance of resistant bacteria in the absence of antimicrobial drug.

## Abbreviations

*E. coli*: *Escherichia coli*
PD: pharmacodynamic
MIC: minimum inhibitory concentration
CFU: colony forming units
MH-2: Mueller–Hinton 2
OD: optical density
PK: pharmacokinetic
